# Effects among healthy subjects of the duration of regularly practicing a guided imagery program

**DOI:** 10.1186/1472-6882-5-21

**Published:** 2005-12-20

**Authors:** Eri Watanabe, Sanae Fukuda, Taro Shirakawa

**Affiliations:** 1 Department of Health Promotion and Human Behavior, Kyoto University Graduate School of Public Health, Yoshida-Konoe-cho, Sakyo-ku, Kyoto 606-8501, Japan; 2 Department of Physiology, Graduate School of Medicine, Osaka City University, 1-4-3 Asahimachi Abeno Osaka 545-8585, Japan

## Abstract

**Background:**

We examined a large number of healthy adults in the general community who had individually participated in a guided imagery (GI) program daily and for various durations, to examine the psychophysiological effects of a GI program within a healthy group.

**Methods:**

We studied 176 subjects who had participated in sessions that were part of a guided imagery program, and who had practiced GI at home for 20 minutes once daily in a quiet place after mastering GI in the group sessions. The average duration of GI practiced at home was 6.88 ± 14.06 months (n = 138, range: 0 to 72). The Multiple Mood Scale (MMS), Betts (1909) Shortened Questionnaire on Mental Imagery (QMI), and a visual analog scale (VAS) of imagery vividness, salivary cortisol (C_S_) levels, general stress and general health were used in the sessions.

**Results:**

We examined the relationship between the duration of daily GI practiced at home and MMS, QMI, C_S_, general health, and general stress at baseline. The subjects who had practiced GI at home longer had lower negative mood scores at baseline and lower severity of stress, and higher positive mood at baseline (both at a session and at home), general health, and QMI scores at baseline. The MMS change during a session and the duration of daily GI practiced at home were not correlated. Repeated-measures analysis of covariance showed that the duration of daily GI practiced as the covariate was not associated with changes in the three C_S _levels.

**Conclusion:**

Although regularly practicing a GI program daily for 20 min did not affect the C_S _level or mood during a GI session for several hours, it kept a good condition of the general mental, physical well-being and their overall stress of the practitioners as they had practiced it for long duration. We postulate that subjects who have the high ability of imaging vividness showed the better mood, health status and less stress than those subjects who have the low ability of it did. The ability of image vividness of the long-term regular practitioners of GI was higher than its short-term or inexperienced practitioners, which allowed practitioners to produce more comfortable imagery. Consequently, the longer the duration that they had practiced GI program once a day regularly, the lower scores of their stress were and the higher scores of their health were. We suggest that the regular daily practice of a GI program might be connected to less stress and better health.

## Background

Guided imagery (GI), a mind-body relaxation technique, is a cognitive, behavioral technique that allows individuals to exert active control over their focus of attention [1,2]. GI has been used to manage various symptoms; it is effective in alleviating chronic pain and reducing headache, and it is beneficial to patients undergoing chemotherapy or to cancer patients in general [3]. We focused on the effects of GI on stress and psychological distress in healthy adults, because we thought that stress reduction in healthy people is difficult using the methods of conventional Western medicine. Many studies have explored GI [4,5], and preliminary evidence supports the effectiveness of GI for managing stress, anxiety, and depression, as well as for lowering blood pressure and reducing pain and the side effects of chemotherapy [3].

We attempted to clarify how the daily practice of a GI program at home or in group sessions influences the mind and body and whether GI effects on health. Therefore, we investigated the effects of the duration of practicing GI individually at home on a daily basis.

We cooperated with a public company that conducted an original GI program for healthy adults for the purpose of reducing daily stress and promoting health care. The company offered group GI sessions to the public once a month, and the therapist published books and audio CDs on how to practice a GI program individually at home [6]. Our previous pilot study investigated GI practiced among healthy members of the community [7]. That study suggested that subjects who had practiced GI program individually at home for long duration and had participated in group sessions frequently had kept lower negative mood scores and salivary cortisol (C_S_) levels and higher positive mood scores and their ability to produce vivid imagery above their baseline levels, than subjects who had practiced it for no or short duration.

Few studies have examined the relationship between the frequency of practicing GI and its outcome [3]. Some studies have indicated that the frequency of practicing GI has no effect on reducing blood pressure [8,9] or depression scores [10]. The relationship between the duration of practicing GI daily and psychophysiological measures remains unclear, due to a lack of research in this area.

Therefore, we examined a large number of healthy adults in the general community who had individually participated in a GI program daily and for various durations, to examine the psychophysiological effects of a GI program within a healthy group. We assumed that investigating a group of subjects who have selected to use our GI program would be more meaningful than an laboratory experimental study, because the effects should reflect their daily lifestyle more accurately.

## Methods

**Figure 1:**
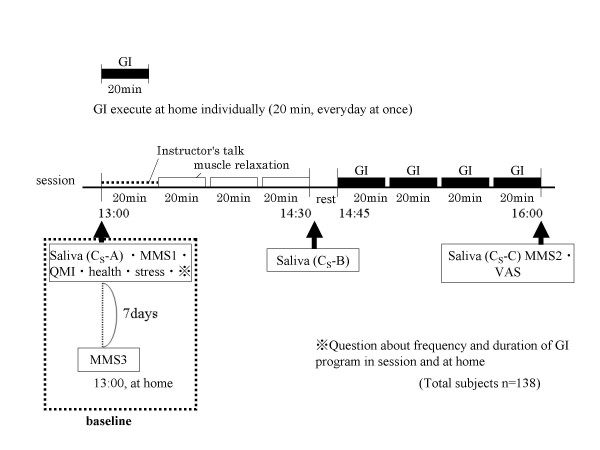
Timetable for the sessions and the experimental procedure.

We investigated the study at a certain public GI group that had been conducted it monthly for several years and studied the participants attending the sessions (see Figure 1). The participants were healthy adults who had learned about the sessions after reading books about GI written by a therapist [6]. The age, gender, occupation, address, and individual attributes varied among the subjects. They had participated in the GI sessions voluntarily to learn how to reduce stress at work and maintain their health status. The participants were not specifically sought out for this study, because we investigated these subjects at public GI sessions that took place regularly once a month. We explained the purpose and procedure of our study to the participants individually prior to a session. Of 176 session participants who were invited to participate in our study, 28 people declined. Therefore, we examined 148 of the 176 session participants (84%). Participants included 50 males (age: 37.43 ± 11.67 years [mean ± SD], range: 18 to 70) and 98 females (age: 41.05 ± 11.57 years, range: 22 to 76). We statistically analyzed complete data on 138 subjects, discarding data for subjects who failed to answer questionnaires, were taking medication, or for whom we experienced technical problems regarding the C_S _analysis. All subjects provided written informed consent. We used ID numbers instead of names to protect personal information when compiling statistics. After completing the questionnaires, the subjects volunteered to provide saliva samples at assigned times.

### Guided imagery (GI) program

#### The GI program of group sessions following with explaing the GI method (Live)

The program schedule is shown in Fig. 1. Each GI program group session lasted 3.5 hours. The session room was kept at a temperature of about 20°C and was illuminated using fluorescent lamps only.

During the first half of a session, muscle relaxation [6] was performed. For muscle relaxation, the subjects were mainly taught how to stretch. The therapist informed the participants that muscle relaxation plays an important role in allowing participants to introduce more comfortable imagery. The participants repeatedly tensed and relaxed each skeletal muscle of the body while practicing abdominal breathing. During skeletal muscle relaxation, the participants were instructed to invoke mental images such as, "My muscles are comfortably stretched". This method is similar to progressive muscle relaxation, but the script was original [6]. In the second half of a session, GI was performed every 20 minutes. The participants closed their eyes while performing abdominal breathing, using a locus or facing upward. A audio CD entitled "Water", comprised of slow-tempo, mixed nature sounds, *e.g*., the sounds of water flowing or birds singing, served as background music. The therapist instructed the participants, following the contents of Table 1. Although the scripts varied, in all cases, the imagery was based on "light". After telling the participants, "You feel relaxed, refreshed, and at ease", the therapist allowed them to open their eyes.

**Table 1:** Scripts used for guided imagery

Imagery		
Sun	Moon	Thanks

1. Close your eyes lightly.	1. Close your eyes lightly.	1. Close your eyes lightly.
2. You are sitting on a white sandy beach along the ocean.	2. A full moon shines on your head, and its light falls on your chest.	2. Think of a family member or friend whom you want to thank.
3. The surface of the sea is shining from the light of the rising sun.	3. The light from the full moon expands to cover your entire body.	3. The light of the rising sun is illuminating your entire body.
4. The light of the rising sun is lighting up your entire body.	4. The comfortable light of the full moon expands to fill this room.	4. This comfortable light is also illuminating that person's entire body.
5. Loving and healing light from the rising sun lights up your head, face, shoulders, arms, chest, stomach, back, hips, thighs, knees, both legs, and toes in turn.	5. This light is yourself, and the light expands to illuminate this building, this town, Japan, and the Earth.	5. You send a message of thanks to that person from your heart.
6. This warm light surrounds your entire body and soaks into each cell.	6. You look at the blue earth from space and feel relaxed.	

* The participants perform this method while breathing from the abdomen. They imagine that anxiety and worry leave when they exhale.

#### GI performed individually at home (with a CD including therapist's voice)

The participants of the group session practiced GI at home after mastering GI in the group sessions. Half of the subjects performed its GI program at home once a day. The individuals incorporated the practice into their daily lives as they saw fit, although the therapist recommended that they recall the imagery while breathing from the abdomen for 20 minutes once daily in a quiet place using a locus or facing upward. The participants used therapist-selected pleasant imagery in their GI programs and chose their favorite imagery from a script (Table 1) [6]. They practiced the GI program at home while listening to a special audio CD, which included the same imagery, with the therapist's voice and background music as in the group sessions. With the CD, the participants could reproduce the content of a group session at home. A set of imagery continued for 20 minutes. The same therapist instructed the participants both in the group sessions and on the CD used individually at home.

**Table 2:** Mean and standard deviation of each measure

		average	p value	SD
MMSPO	1. baseline	36.32	***(1)	7.87
	2. after a session	40.20	***(1)	8.09
	3. at home	38.55		8.15
MMSNE	1. baseline	31.51	***(2)	9.29
	2. after a session	24.66	***(2)	7.66
	3. at home	27.81		8.80
Imagery	QMI	183.13		35.80
	VAS vividness imagery	5.61		2.23
salivary cortisol	A. before a session	0.420	***(3)	0.372
	B. during a session	0.391	***(3)	0.388
	C. after a session	0.288	***(3)	0.280
self-reported	health	2.29		0.53
	severity stress	2.15		0.89

(1)(2) paired t-test (3) Repeated-measures ANOVA analyses. The changes in the MMS score and CS level in the sessions were significant. *** p < 0.001

PO; Positive mood, NE; Negative mood

To record the frequency and duration with which GI was performed at home, we asked the subjects following questions. "How many times have you performed GI program by yourself at home within these 30 days? Select appropriate alphabet and write a number. A) No experience. B) more than once a month and less than once a week; How many times a month? C) more than once a week and less than once a day; How many times a week? D) more than once a day" "How long duration have you performed GI at home?"

### Saliva sampling and CS assay

We collected saliva samples using a plastic tube that contains cotton (Salivette; Sarstedt, Rommelsdorf, Germany). To collect samples, the subjects chewed on the cotton for 90 sec. The samples were centrifuged for 5 min at 3,000 rpm at 4°C immediately after collection and were stored at -20°C until they were assayed in duplicate. The saliva cortisol (C_S_) levels were measured using a radioimmunoassay method with a GammaCoat™ cortisol kit (DiaSorin, Mitsubishi Kagaku BCL, Japan) [11].

### Psychological measures [7]

#### Short form of the Multiple Mood Scale (MMS) [12]

Mood states were measured using a mood adjective checklist constructed in Japan; its reliability and validity have been confirmed in studies of Japanese undergraduate students. It has been used widely in Japan and can be used for a general population more than teenager from its contents [13]. The scale has eight subscales, with five items each, representing depression (anxiety), hostility, and boredom as negative (NE) moods, liveliness, well-being, and friendliness as positive (PO) moods, and concentration and being startled as relatively neutral moods. We measured the PO (the sum of the liveliness, well-being, and friendliness scores) and NE (the sum of the depression, hostility, and boredom scores) mood scores. MMS can change in several hours. The internal consistency of MMS had been tested instead of test-retest reliability (α = 0.83–0.92) [12].

#### Betts' (1909) Shortened Questionnaire on Mental Imagery (QMI) [14]

Sheehan (1967) published a shorter 35-item version of the QMI, which was found to correlate highly with the complete Betts' QMI. The validity of the long form has been demonstrated by high correlations between QMI scores and actual ability to generate images in experimental settings [15] A Japanese version of the QMI was constructed [16] and Tabane's version was used in our study. Reliability and validity of this version have been well demonstrated in populations of university students [16]. The short QMI is a 35-item questionnaire designed to measure the ability to generate images using seven modalities: visual, auditory, cutaneous, kinesthetic, gustatory, olfactory, and organic, for which subjects recorded their responses using a seven-point vividness rating scale. In this study, the participants were instructed to rate how clearly each suggested image appeared to them on a scale of 1 (no image at all) to 7 (perfectly clear). Higher scores indicate a greater ability to generate mental images. In the resent study, the internal consistency of QMI is α = 0.97. [17].

The vividness of the imagery was measured using a visual analog scale (VAS, 0–100 mm) [18] to assess whether the subjects were able to remember imagery clearly, according to instructions, during the course of a session. The subjects rated image vividness along a 100-mm measuring stick, anchored at the left by the phrase "no image at all", and at the right by the phrase "perfectly clear". Such VAS questions of vividness imagery was used on the Tobacco Craving Questionnaire (TCQ), and have been verified the reliability and validity of the TCQ and VAS. Test-retest reliability of VAS question on TCQ was tested and it ranged r = 0.19–0.28 [19].

General stress severity (three levels) and general health (four levels) were converted into point scores (stress: little = 1 point, some = 2, much = 3; health: very good = 1, good = 2, bad = 3, very bad = 4). The test-retest reliability of the stress and health states was not tested, but the similar scales, Perceived Stress Scales (PSS), had been tested. The test-retest reliability of PSS over 6 weeks was 0.55 in resent study [20]. The questionnaire also asked for the alcohol and smoking status, as well as the behavioral style (their hobby, sleep time, and other exercises) of a subject by the free form. The subjects responded to all the psychological measures in a written questionnaire.

### Procedure

To examine the usual states of the subjects, we took saliva samples to measure cortisol (C_S_-A) and collected the completed questionnaire, which included the MMS1, QMI, health, stress, individual GI practice (duration and frequency of GI practice at home, frequency of group session participation), and other attributes as a baseline before a group session. In addition, the usual mood state of the subjects at home was determined from a written questionnaire (MMS3), administered 1 week after the sessions. These questionnaires were collected by mail. To examine changes when the subjects practiced GI, we collected saliva samples to measure cortisol during (C_S_-B) and after (C_S_-C) the session and collected the completed questionnaire on MMS2 and VAS vividness imagery after the session, using the schedule shown in Figure 1.

### Statistical analysis

To examine change of measures itself, paired *t*-test was used to test the changes in the MMS scores and repeated measures analysis of variance was used to test the changes in the Cs levels.

The duration of GI practiced at home was not normally distributed, because more than half of the subjects did not practice GI at home (n = 78). To examine the relationship between the duration of GI practiced at home and the psychophysiological measures {MMS1, MMS2, change scores of MMS (MMS2-MMS1), QMI, VAS scores, and C_S _levels}, Spearman correlation coefficients were calculated as a nonparametric test. Repeated-measures analysis of covariance (ANCOVA) -were used to test the changes in C_S _levels and to examine the duration of GI practiced at home or potential covariance factors related to changes in the C_S _levels. To compare duration of their GI execution in the same condition; practicing GI once a day, the experienced subjects who did not practice GI at home irregularly (n = 11) were excluded from analysis, giving 127 subjects who practiced GI regularly, including inexperienced subjects. Because we extracted subjects who had in condition definitely. The analyses were performed using the Statistical Package for the Social Sciences (SPSS) ver10J.

## Results

The mean and standard deviation of each measure are shown Table 2. The changes in the MMS scores and C_S _levels differed significantly, as reported previously [7].

### The frequency and duration of GI in group session or at home in these subjects

The average frequency of participation in these subjects to the GI group sessions before this investigation was 3.09 ± 4.22 sessions (n = 138, range: 1 to 25).

The frequency of GI practiced at home ranged from subjects who did not practice GI at all to subjects who practiced it daily: not at all (n = 78), once per week (n = 3), twice per week (n = 2), three times per week (n = 3), four times per week (n = 2), five times per week (n = 1) and seven times per week (n = 49). Nobody selected B)"more than once a month and less than once a week" or D)"more than once a day". All subjects who participated the group session for the first time selected A)"No experience". All subjects who participated the group session more than two times selected C)"more than once a week and less than once a day", and most of them had practiced everyday as the therapist encouraged.

The average duration of GI practiced at home was 6.88 ± 14.06 months (n = 138, range: 0 to 72). The duration of GI practiced at home and the frequency of participation in GI group sessions were highly correlated (Spearman's correlation coefficient, R = 0.741, *p *< 0.001)

### Effects related to the duration of daily GI practices at home

#### Baseline

We examined the relationship between the duration of daily GI practiced at home and MMS, QMI, C_S_, general health, and general stress at baseline (Table 3). The subjects who had practiced GI at home longer had lower NE mood scores at baseline and lower severity of stress, and higher PO mood at baseline (both at a session and at home), general health, and QMI scores at baseline.

**Table 3:** Correlation between the duration of daily practicing GI individually at home and baseline measures (MMS, QMI, CS, health and severe stress) (n = 127).

correlation to GI duration (Spearman)			
Measure		R	*P*

MMSPo	1 baseline	0.193	0.042*
	3 seven days later	0.218	0.044*
MMSNe	1 baseline	-0.250	0.008**
	3 seven days later	-0.196	0.120
QMI		0.194	0.042*
Salivary cortisol (A)		0.060	0.521
self-reported	health	-0.236	0.010*
	Severe stress	-0.183	0.048*

*p < 0.05, **p < 0.01, Spearman correlation coefficients

Baseline was measured before session (13:00).

"Seven years later"; Seven days later of a session, MMS was measured individually at home (13:00).* The experienced subjects who did not practice GI at home everyday were exclude (n = 11)

#### Changes when subjects practiced GI in sessions

Although the baseline differed, the MMS change during a session (the change in the NE and PO mood scores, MMS2-MMS1) and the duration of daily GI practiced at home were not correlated. Of the other measures, the MMS change during a session and VAS vividness imagery were correlated (PO mood change, R = 0.187, *p *= 0.035; NE mood change, R = 0.244, *p *= 0.005). Repeated-measures analysis of covariance (ANCOVA) showed that the duration of daily GI practiced as the covariate was not associated with changes in the three C_S _levels (F = 0.233, *p *= 0.792). Of the other measures, age and QMI were strongly connected to the changes in C_S _levels when we used various measures as covariates, such as age, gender, alcohol states, smoking status, sleep time, eating, stress, health, MMS, QMI, VAS, and individual GI practice (age; F = 7.229, *p *= 0.001, QMI; F = 2.684, *p *= 0.070). We found that a longer duration of daily practice was correlated to higher VAS vividness imagery in the session scores (R = 0.338, *p *< 0.001).

## Discussion

This study investigated the effects of the duration of regularly practicing a GI program at home. The results partly concur with those of our pilot study. Our results suggest that subjects who had a longer history of practicing a GI program at home once daily for 20 min showed higher baseline scores of their positive mood on MMS, image vividness on QMI and general health, and lower baseline scores of their negative mood on MMS and general stress than subjects who had shorter or no history of GI at home. The longer the subjects had practiced GI on their own, the higher their VAS vividness imagery scores were after the GI group session. It is supposed that subjects, who practice GI at home for a long time, can produce imagery more vivid than beginners or the subjects for a short time can when they are guided by a therapist in a group session. Our findings regarding the NE mood scores concur with a cohort study that examined organ-transplant patients who practiced mindfulness-based stress reduction at home (45 minutes, 5 days a week) and showed the related to that their low anxiety scores significantly [21].

The change in the MMS score from baseline during a session was not correlated to the duration of practicing GI at home daily. Although an earlier study reported that the change in the C_S _levels differed significantly with the duration of GI practice [22], our ANCOVA indicated that the change in the C_S _levels at three points during a session was not correlated with the duration. Rather, the MMS mood change was significantly correlated to the VAS vividness imagery score, and the change in the C_S _level was significantly related to the QMI scores. As for the image vividness measured using the QMI, a cross-sectional study comparing subjects with poor and vivid imaging abilities reported that high-ability imagers had lower stress levels and fewer negative moods after listening to a relaxation tape than low-ability imagers[23]. Based on the report by Johnsen (2001) and our results, we postulate that daily practice of GI allows practitioners to develop more vivid images over the long term, which results that long practitioners of regular GI at home had kept more positive mood and the lower stress than short practitioners, but it cannot be proved by effects only of this study. Imagery vividness may thus be the key to the outcome of GI [24].

Several limitations of our study warrant consideration. (1) A muscle relaxation session, abdominal breathing, and the background music used to facilitate imagery are all thought to be effective in themselves. We must therefore recognize the effects of a GI program as a whole, not just the effect of imagery itself. (2) The duration of practicing GI at home and the frequency of participating in GI group sessions were highly correlated. When we consider the effects of the duration of regular practice of GI, we cannot exclude the effects of frequent participation in GI group sessions. Session participation once every few months at most will play a role in learning the correct method of GI and motivating people to practice GI regularly. It is difficult to conceive how the group sessions affect the daily baseline of the mind and body. (3) We assumed that the QMI remained constant over a period of several hours, but need to confirm whether the scores change after practicing a GI program. (4) The data on the duration and frequency with which subjects practiced GI at home depended on their memory, and from 1 month to several years had passed since they had started practicing GI at home. We could not verify the accuracy of these data. (5) The subjects who enjoyed practicing GI likely continued their program for a longer period. We must compare the results of this study to an appropriate experimental study that includes controlled conditions. (6) C_S _baseline level was not significantly related to duration of GI practice everyday at home. The reason is may be baseline of C_S _level was different by age [25]. Our future study need to be controlled about age factor.

Our results suggest that practicing a GI program once daily for 20 min results in keep positive mood, good health and high ability of vividness imagery. Therefore, we postulate that the subjects who produced specific images, such as light and nature, could relax their mind and body, thereby reducing stress and maintaining their health.

## Conclusion

Although regularly practicing a GI program daily for 20 min did not affect the C_S _level or mood during a GI session for several hours, long practitioners had kept the better general mental and physical well-being and less overall stress than short or inexperienced practitioners. We postulate that the ability to produce vivid images is the connection between guided imagery and the positive mood, good health status and less stress. Image vividness may be creatured by the long-term regular practice of GI, which allowed practitioners to produce comfortable imagery more effectively. Consequently, stress and health of long practitioners supposed to keep good condition as compared to short or inexperienced practitioners. We suggest that regular daily practice of a GI program have a possibility to confer benefits in reducing stress and improving health.

## Competing interests

The author(s) declare that they have no competing interests.

## Authors' contributions

EW carried out all of the experiments and participated in drafting of the manuscript, SF coordinated the study and participated in the design of the overall project and the data analyses and TS supervised the work of EW and SF and helped to obtain funding. All authors read and approved the final manuscript.

## Pre-publication history

The pre-publication history for this paper can be accessed here:


